# Positive behaviour support: a systematic literature review of the effect of staff training and organisational behaviour management

**DOI:** 10.1080/20473869.2022.2123199

**Published:** 2023-02-01

**Authors:** Ioanna Konstantinidou, Karola Dillenburger, Devon Ramey

**Affiliations:** Centre for Behaviour Analysis, Queen’s University Belfast, Belfast, UK

**Keywords:** Positive behaviour support, applied behaviour analysis, staff training, challenging behaviour, organisational systems, knowledge, skills, or behaviour change

## Abstract

Positive Behaviour Support is an applied behaviour analytic system of support that is utilised in schools and in residential care settings for children and adults with disabilities who engage in challenging behaviour. Implementation fidelity depends on appropriate staff training and organisational behaviour management. A systematic literature review is reported that evaluated the evidence in relation to change in staff and service user behaviour and the impact of organisational behaviour management systems on effectiveness, generalization, and maintenance of these outcomes. Nine relevant articles were identified and analysed according to (1) the demographics of staff and residents and methods of staff training; (2) organisational behaviour management systems; (3) staff and service-user behavioural outcome measures; and (4) the methodological quality of the study. A combination of antecedent and consequence-based training strategies was used in the studies. Eight studies reported on the organisational behaviour management systems that were used, with five reporting on the responsibility of trainees to transfer their training to their untrained teams (pyramidal training). Although the studies reported on staff behaviour change following the training, only one of the studies reported significant increases of service user quality of life as a result of staff training and only two studies provided adequate methodological strength.

## Introduction

Positive behaviour support (PBS) is a multicomponent framework for supporting people with intellectual disabilities who engage in behaviour commonly described as challenging (Gore *et al.*, 2013). PBS is one of many applications of the science of behaviour analysis (NICE, 2015). Like other natural sciences, behaviour analysis has three interconnected branches: the conceptual analysis of behaviour (radical behaviourism), the experimental analysis of behaviour (EBA), and the application of the science, applied behaviour analysis (ABA) (Cooper *et al.*, 2019). ABA is ‘the science in which tactics derived from the principles of behavior are applied systematically to improve socially significant behavior, and experimentation is used to identify the variables responsible for behavior change’ (Cooper *et al.*, 2019, p.3). PBS utilises ABA-based procedures to focus on

- developing a functional assessment of the social and physical context within which challenging behaviour occurs; including direct behavioural observations, interviews, record reviews, and behaviour rating scales;
- the inclusion and involvement of key stakeholders, including the family, friends, other family members, care staff and/or therapists;
- developing, implementing, and evaluating the effectiveness of comprehensive person-centred systems of support aimed at enhancing the quality-of-life of the person using procedures including
- a clear description of the targeted behaviour, triggers or antecedents of the behaviour, maintaining consequences, and the function of the problem behaviour,
- strategies to reduce the probability of the problem behaviour, including environmental arrangements, personal support, changes in activities, prompts, and changes in expectations,
- teaching of skills to replace the problem behaviour, use positive reinforcement for promoting appropriate behaviour, and ensuring behaviour generalizes and is maintained via developing friendships and getting involved in the community (Gore *et al.*, 2013, p.15; NCPMI, 2022).

In concord with disability rights and relevant government guidelines, PBS focuses on value-based, theory-driven, evidence-base practice (Beadle-Brown and Murphy, 2016, Denne, 2015). The quality of PBS services depends to an important extent on the skills of front-line staff (Reed and Henley 2015). While staff in education settings usually are teacher trained or receive training as teaching assistant, commonly, frontline staff in residential care settings do not hold recognised professional qualifications. Residential staff usually receive in-house, on the job training, either specifically in PBS philosophy and procedures or more generally in the overarching scientific discipline of ABA (Gormley *et al.*, 2020). The quality and effectiveness of this training and the organisational behaviour management (OBM) systems in place to ensure intervention fidelity directly affect services user outcomes (Reed and Henley, 2015), including their social engagement (Szczech, 2007) and quality of life (Jahr, 1998). OBM applies behaviour analytic knowledge to performance management, system analysis and behaviour-based safety (Agnew and Uhl, 2021, Daniels, 2000, Daniels and Bailey, 2015).

The traditional ‘train and hope’ approach (Stokes and Baer 1977) is not effective and can even have a negative impact on service delivery (Campbell 2007). In contrast, there is ample evidence that behavioural skills training can be effective (Campbell 2010; Griffin *et al.*, 2019). However, a number of barriers remain, such as a lack of measures to ensure intervention fidelity and a failure to ensure generalization of new skills across the team (Smith *et al.* 1992). Consequently, MacDonald *et al.* (2018) identified the need for a ‘whole organization’ approach to staff training. This approach does not only include behaviour skills training for individual staff, it also includes the identification of effective OBM processes to ensure training leads to the desired outcomes.

The impact of staff training on behavioural skills has been explored in a number of reviews (Gormley *et al.* 2020, MacDonald and McGill 2013, Shapiro and Kazemi 2017) and meta-analyses (van Oorsouw *et al.* 2009). Most of these studies focused on teaching specific behavioural intervention procedures, rather than educating staff in an overarching scientific framework. For example, Shapiro and Kazemi (2017) reported that training in token economy or discrete trial teaching (DTT) resulted in increased staff skills in these procedures, meeting mastery criteria in 18 of the studies. Gormley *et al.* (2020) reported a scoping review of studies regarding antecedent-based procedures and found that most studies reported improved staff skills with regards to these procedures. Van Oorsouw *et al.* (2009) conducted a meta-analysis and concluded that overall staff training was effective.

In sum, while the effectiveness of staff training in commonly used procedures is relatively well documented, outcomes for clients regarding quality of life or reduction of challenging behaviour are reported less often (MacDonald and McGill 2013). Furthermore, little is known about the OBM structures used to support effective long-term outcomes of staff training. The objective of the present systematic literature review was to analyse outcomes of staff training in relation to overarching frameworks (i.e. PBS and/or ABA) and service-user outcomes and to assess the impact of different OBM systems. Given that Dickinson (2001) covered the historical roots of OBM earlier, the present review covered studies published since then.

## Methodology

**Figure 1.**
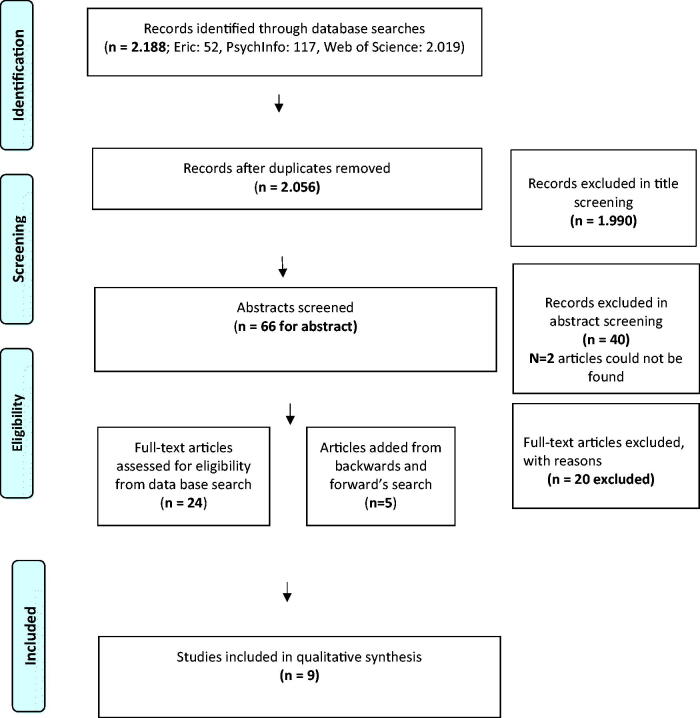
Prisma chart presenting the screening process.

The search methods used in this review were based on the Preferred Reporting Items for Systematic Reviews and Meta-Analyses (PRISMA) framework and findings are reported according to the PRISMA checklist (Moher *et al.* 2009) (Figure 1).

### Inclusion and/exclusion criteria

To be included in this review, the study had to be conducted within a social care setting (including residential services, community-based programmes, training providers for disability services) for adults with intellectual disabilities and

1. be peer-reviewed and published in English between 2000 and 2021;
2. describe staff training in PBS and/or ABA, as the independent variable;
3. report on outcomes for at least one of the following: quality of staff skills, quality of behaviour support plans, and fidelity of implementation of intervention.

Studies were excluded if

1. the independent variable of the study was training on only one specific behavioural procedure (e.g., Discrete Trial Training; most-to-least prompting);
2. they described school-wide PBS or training for staff in formal school settings;
3. they described training for staff only in specific professional roles e.g., social workers, speech and language therapists (unless the training met inclusion criteria);
4. they described training of staff who already had qualifications in ABA, for example, a degree in behaviour analysis;
5. they included parents training;
6. they were systematic reviews, process reviews, and grey literature.

### Screening procedures

Searches of electronic databases (ERIC, PsycINFO, Web of Science) were conducted in June 2021. The following search terms were used: ‘positive behavio* support’, ‘PBS’, ‘applied behavio* analysis’, ‘ABA’, and ‘staff train*’. The terms were combined using variations of Boolean operators, according to each database. Specifically:

1. for Eric (‘positive behavio* support’ or pbs) AND staff N3 train* and (‘applied behavio* analysis’ or aba) and staff N3 train*,
2. for PsychInfo (‘positive behavio* support’ or pbs) AND staff adj3 train* and ‘applied behavio* analysis’ or aba) AND staff adj3 train*,
3. for the Web of Science: ‘applied behavio* analysis’ or aba and ‘staff NEAR/3 train*’ and ‘positive behavio* support’ or pbs and ‘staff NEAR/3 train*’.

The search resulted in 2188 articles (Eric: 52, PsychInfo: 117, Web of Science: 2019). After removing all duplicates, a total of 2056 studies remained. Title screening excluded 1990 studies. Of the remaining 66 studies, 42 were excluded during abstract screening, including 2 studies that did not have an abstract. This resulted in 24 studies eligible for full text screening. Forward and backwards searches were conducted, including searching for additional published research by the first author of these 24 studies, searching the reference sections of these 24 articles, and searching Google Scholar. An additional five studies were identified. Therefore, full text screening was conducted for 29 studies and this resulted in the exclusion of a further 20 studies. Nine studies met all the inclusion criteria (Figure 1).

### Inter-rater reliability

Inter-rater reliability checks were conducted by the first author as well as another Board-Certified Behaviour Analyst. Inter-rater reliability was assessed for 50% of the studies that were eligible for full text screening (inter-rater reliability = 93.33%.). Inter-rater reliability also was calculated for the data extraction of all categories in 30% of the studies. This resulted in 100% inter-rater reliability for the general and training information, 90% inter-rater reliability for the outcomes, and 80% inter-rater reliability for organisational systems. For the quality assessment of the studies, the criteria were discussed with a second rater until 100% agreement was achieved. The IOA for the quality assessment was 93.33%.

### Categories for analysis

Apart from general and demographic information about staff training (i.e. participants, setting, type of training, length of training, country in which the study took place) information was extracted for behavioural outcomes (staff and service users) and organisational systems.

Behavioural outcome categories for staff included their knowledge (i.e. theoretical, factual, procedural, value-based) and skills (i.e. practical, interpersonal, technical, problem-solving), the overall quality of support they provided including the fidelity of implementation of the interventions, and the quality of the behaviour support plans they designed. Information on other outcomes including staff attitudes and beliefs were also provided.

The measurement systems used for the quality of staff skills, quality of behaviour support plans and fidelity of implementation of interventions were also included. All outcomes for residents were analysed, regardless under which category they were reported in an article.

OBM systems were categorised according to Gravina *et al.* (2018) into those that used antecedent-based strategies and those that used consequence-based strategies. Antecedent-based strategies were strategies that were in place before or at the beginning of the training and included:

1. Assessment (Assess), such as collecting and using data to identify training needs or OBM systems in place to reinforce training outcomes;
2. Training for staff in supervisory roles (super.roles);
3. Clarification of responsibility for generalization of learning across the whole team (respons.), such as train the trainers model or training to implement of specific training goals;
4. Other antecedent-based strategies that were implemented prior to or at the beginning of training, which do not meet criteria for the categories described above. For example, ‘prompts’ or other supports to reinforce the training and the desired outcomes or ‘change in resource availability’ (for example, reducing trainees’ responsibilities).

Consequence-based strategies were those that were in place as part of longitudinal training or after the completion of staff training. These included

1. Monitoring/feedback systems (mon/feed systems) that were in place continuously to evaluate training outcomes and systems to deliver performance feedback after the training. Monitoring systems that were used only by the researcher of the study or external trainers were not included;
2. Other consequence-based interventions in place to reinforce implementation or generalization of training outcomes.

Brief information about the effectiveness of each study regarding the target outcomes was provided. Outcomes were scored as positive, if they led to a significant change. Information was also provided for the studies that reported results on maintenance (i.e. after the post-training phase) and generalization of the staff skills to the staff group.

The overall quality of the studies was assessed using Reichow *et al.*’s (2008, Reichow et al 2011) evaluative method for determining evidence-based practices (EBP) in autism. Reichow *et al.* propose three levels of assessment:

1. rubrics for the evaluation of research report rigor;
2. guidelines for the evaluation of research report strength; and
3. criteria for the determination of EBP.

Research rigor assessment was similar for group research and for single subject research. Common primary quality indicators included participant characteristics, dependent measures, and independent variables; common secondary quality indicators including interobserver agreement, blinding of raters, procedural fidelity, generalization and maintenance, and social validity. Research strength was assessed on three levels: strong (provide concrete evidence of high quality), adequate (showing strong evidence in most, but not all areas), and weak (many missing elements, and/or fatal flaws). Finally, criteria for the determination of EBP included two categories: established EBP and promising EBP. Established EBP were effective across multiple strong studies conducted by at least two independent research groups, while promising EBP were effective across multiple studies but there was some evidence that practice was limited by weak methodological rigor, few replications, or an inadequate number of independent researchers demonstrating the effects (Reichow *et al.* 2008)

## Results

### General information

The studies reviewed included a total of 876 members of staff, including managers and frontline staff, as well as 202 adults with intellectual disabilities (Table 1). The training was carried out mainly in large-scale statutory and voluntary sector residential settings, located in the community or in hospital settings. The main training methods were pyramidal style ‘train the trainer’, with some of the programmes focussing on staff skills development while others focussed on system-wide interventions. Most of the training was relatively short-term, over fewer than 5 days, while some of the maintenance training stretched over 9-12 months.

**Table 1.** General and training information.

Authors	Participants	Setting	Country where the study took place	Training	Duration
Crates and Spicer 2012	3 first generation trainees Second generation trainees 32 clients (25 adults, 7 children)	Disability services in Tasmania	Australia	Train the trainer model: Trained trainers who attended courses provided by the Clinical Director of the Institute for Applied Behaviour Analysis (IABA) (LaVigna *et al.* 2005). The training was replicated in 2006 by the Tasmanian training team to second generation trainees. Tasmanian trainers were prepared, supervised and monitored by IABA. PBS course following IABA’s model (LaVigna *et al.* 2005) Level 1 (including topics such as IABA’ s multi-element model, functional behavioural assessment, Level 2 (longitudinal Practicum)	4 days (Level 1) 9 days spread over a period of 9 months (Level 2)
Dench 2005	38 students/staff	St. John of God Hospitaller Services (for people with learning disabilities)	UK	Training in Multi-Element Behavioural Support, adapted from IABA, and based on the intensive intervention sequence (Donnellan *et al.* 1985) Supervisors’ workshop for all staff attending training	9 months
Haberlin *et al.* 2012	44 direct care staff, (after excluding those not meeting criteria for analysis, 10 staff remained from the consultant-led training and 16 from pyramidal training. 4 supervisory staff	Community based programmes	USA	PBS course delivered either through pyramidal training or through consultant led training Most of the materials came from the manual Positive Behavior Support Training Curriculum: Supervisor Edition (Reid and Parsons 2004) In pyramidal training the supervisors were trained by the consultants	Consultant-led training: 5 training sessions (1 h each) Pyramidal training: 6-7 one-hour sessions within 2.5 weeks. Supervisors: 5 training sessions (1 h each) + 1 h training on how to deliver feedback
MacDonald *et al.* 2018	72 managers 72 clients Staff managed by managers	Community based social care provider	UK	PBS course ‘multielement model’ (Lavigna and Willis 2005)	1 year (2 days introduction, 8 one-day workshops, 6 weeks apart)
McGill *et al.* 2018	81 adults with intellectual disabilities Secondary participants: staff and managers	Residential services for adults	UK	Training as part of system wide intervention	11 months intervention
McKenzie *et al.* 2002	36 staff	Non-statutory residential services	UK	Training on challenging behaviour (described as using the multi-element model)	1 day
O’Dwyer *et al.* 2017	54 staff 17 adults with disability	Agencies registered with the Victorian Department of Human Services	Australia	PBS course included elements of good practice as recommended in the BSP QE-II guide (recently	4 days in two-day blocks conducted 2 weeks apart 12 months (after training for developing an implementation plan for their team)
Reid *et al.* 2003	12 supervisors (1st training for a part of the training curriculum) 386 supervisors	Residential Agency in North Carolina	USA	PBS course following the Carolina Curriculum: The curriculum included supervisory skills, developed under the auspices of the South Carolina Department of Disabilities and Special Needs	5 days within 5 consecutive weeks
Wardale *et al.* 2014	234 staff working in the disability sector	Training course run by the Centre of Excellence for Behaviour Support (training provider)	Australia	PBS course. The Functional Assessment and Positive Behaviour Intervention (FAPBI) training course	4 days course delivered across 6 to 8 weeks

### Staff skills outcomes

The impact of training on staff skills (Table 2) was assessed through the quality of behaviour support plans in four studies (Crates and Spicer 2012, Dench 2005, O’Dwyer *et al.* 2017, Wardale *et al.* 2014), the implementation of behavioural strategies and/or behaviour support plans in four studies (Crates and Spicer 2012, Dench 2005, MacDonald *et al.* 2018, McKenzie *et al.* 2002), the quality of support in two studies (MacDonald *et al.* 2018, McGill *et al.* 2018), and through other competency assessments in two studies (Haberlin *et al.* 2012, Reid *et al.* 2003).

**Table 2.** Type of outcomes and measurement systems for staff overt behaviour.

Authors	Staff skills	Knowledge	Residents’ outcomes	Other outcomes
Crates and Spicer 2012	Quality of behaviour assessment and intervention plan (Assessment and Intervention Plan Evaluation Instrument, AIEI (LaVigna *et al.* 2005); Implementation of strategies in the intervention plans (fidelity) (PSR) Quality of reports reached the standards of the previous training (mean AIEI score for the participants trained by the Tasmanian trainers was 79.5% with a range of 44%–94% for Tasmania 2006, and a mean AIEI score of 80.2% with a range of 64%–92% for Tasmania 2008) The mean PSR score was 47% with a range of 19%–86%		Challenging behaviour 27/30 cases demonstrated a reduction in ES of target behaviour at the 3-month follow-up (statistically significant reduction)	Referral data Cost effectiveness There was a reduction in priority of referrals from 100% Priority 1 in the first year of training provision to 25% Anecdotal reports indi- cated that reduction of challenging behaviour led to related cost savings e.g. resulting from a reduction in support hours, staff injury costs, property damage, and administrative costs associ- ated with issues such as complaints management and grievances
Dench 2005	Quality of behaviour assessment and intervention plan, Callan Institute adapted competencies from the Behaviour Assessment Report and Intervention Plan Evaluation Instrument (Willis and LaVigna 1990) For supervisors: intervention fidelity (PSR) Students achieved an average of 91.7% across all units (the success criterion was 85%) Results of PSR are not described		Quality of life Challenging behaviour The Quality-of-Life Questionnaire (Schalock and Keith 1993) appeared insufficiently sensitive to measure some of the anecdotal evidence 56% cases presented with significant improvement of challenging behaviour (defined by reduction to less than 30% of baseline) within three months of implementation	Organisational cost benefit analysis A culture of positive behaviour support in the organisation. Many of the trained staff have been promoted to key managerial positions and continue to advocate and implement positive behaviour support strategies. There were trained behavioural practitioners in all the services. There were indications that staff make a longer-term commitment to the organisation, reducing staff turnover. The course improved response times and results. The course was an accredited programme, recognised both nationally and internationally
Haberlin *et al.* 2012	For direct care staff: Correct teaching procedures (Checklist with 6 components) For both groups of direct care staff, percentage of correct teaching increased (for pyramidal training group more)	Both groups increased knowledge		
MacDonald *et al.* 2018	For direct care staff: Quality of staff support (momentary time sampling, The Active Support Measure, ASM, completed through observations (Mansell *et al.* 2005); For both direct care staff and managers: Fidelity of PBS plans and staff performance (PSR) (LaVigna 1994) No significant improvement for staff and manager measures	Significant increase in knowledge for managers for the experimental group	Quality of life Challenging behaviour No significant increase for quality of life Significant reduction in challenging behaviour	Staff challenging behaviour attribution Practise leadership for managers No significant increases for both measures
McGill *et al.* 2018	Quality of staff support (momentary time sampling, The Active Support Measure, ASM, completed through observations (Mansell *et al.* 2005) ASM scores increased in 7/9 settings Mean percentage of engagement increased but not significantly		Quality of life Challenging behaviour There was no significant difference between the groups in measurement of quality of life Reduction in challenging behaviour reduced significantly more in the experimental group	Provider’s quality of support Achieved median percentage in last data collection in each setting 80.1%
McKenzie *et al.* 2002	Staff practice (PSR) (LaVigna 1994) The PSR score increased from 26 percent pre-training to 74 percent at 16 weeks after training	Staff rated their knowledge levels as higher after training		Staff challenging behaviour attribution No significant changes in attributional dimensions
O’Dwyer *et al.* 2017	Behave support plan quality (The Behaviour Support Quality Evaluation-II (BSP QE-II) (Browning-Wright *et al.* 2006) The behaviour support quality increased (statistically significant improvement from control group) The quality of the BSPs of the trained group remained below optimal levels		Challenging behaviour Mental health No significant change for severity of challenging behaviour Statistically significant change in client mental health	Frequency of restraints No significant change with the use of chemical restraint
Reid *et al.* 2003	Competency and performance-based observation and training skills (assessed via checklist during direct observation in classroom setting using scenarios for role play and, on the job for reaching mastery criterion) 85% successfully completed the training by performing all classroom and on-the-job skills checks at mastery criterion			
Wardale *et al.* 2014	Skills in developing PBS plans /behaviour support plan quality (The Behaviour Support Plan Quality Evaluation Guide, Version II, BSPQEII) (Browning-Wright *et al.* 2006) Positive results from PSPs quality	Significant change in staff knowledge		Staff challenging behaviour attribution Staff evidence-based practise attitude Significant change in causal attributions No significant difference with evidence-based practises

The studies that reported on the quality of behaviour support plans, used three different evaluation systems. Crates and Spicer (2012) used the Assessment and Intervention Plan Evaluation Instrument (AIEI; LaVigna *et al.* 2005). Dench (2005) used the Callan Institute version of the Behaviour Assessment Report and Intervention Plan Evaluation Instrument (Willis and LaVigna 1990). O’Dwyer *et al.* (2017) used direct observations via the first author assessing the behaviour support plans prior and 12 months post staff training, while Wardale *et al.* (2014) used observations by facilitators after the training without collection of pre-training data. Only two of the studies used the same assessment tool; The Behaviour Support Plan Quality Evaluation-II (BSP QE-II; Browning-Wright *et al.* 2006). Three studies included two outcome measures each (Crates and Spicer 2012, Dench 2005, MacDonald *et al.* 2018).

To evaluate the fidelity of implementation of behaviour strategies and/or intervention plans, four of studies used the Periodic System Review (PSR; LaVigna 1994). For example, MacDonald *et al.* (2018) used the PSR to evaluate the performance for both direct care staff and their managers. McKenzie *et al.* (2002) used specific tasks to assess fidelity for 14 trainees prior to training as well as 16 and 20 weeks after the training.

The quality of staff support was reported in two studies (MacDonald *et al.* 2018, McGill *et al.* 2018) using the Active Support Measure (ASM; Mansell *et al.* 2005). The ASM is an observer completed rating scale, that provides ratings for staff support during activities and choice making. Data were collected during baseline and 3-6 months follow-up.

Other competence-based skills were reported in two studies. Haberlin *et al.* (2012) reported percentage of correctly applied teaching procedures for direct care staff, after training their supervisors, while Reid *et al.* (2003) reported on 26 sets of skills of supervisors. In both cases, checklists were used during observations to record the competence on the relevant skills. In both studies staff skills were assessed on the job, while Reid *et al.* (2003) also assessed the target skills during role play.

Most studies that explicitly focussed on staff outcomes reported improvements. Crates and Spicer (2012) found the quality of reports reached a mean AIEI score of 79.5% (range 44%-94%) for training in 2006, and a mean AIEI score of 80.2% (range 64%-92%) in 2008. Their mean PSR score was 47% (range 19%-86%). Dench (2005) reported that trainees achieved an average of 91.7% across all units (the success criterion was 85%) while PSR were not reported. Haberlin *et al.* (2012) found that for both groups of direct care staff, percentage of correct teaching and knowledge increased, although it improved more for the pyramidal training group. These gains were maintained at 3-months follow up. MacDonald *et al.* (2018) reported no significant improvement in skills for staff and managers, however, knowledge increased significantly for managers in the experimental group.

McGill *et al.* (2018) reported positive ASM scores increased in 7/9 settings and mean percentage of engagement increased, but not significantly. McKenzie *et al.* (2002) found that PSR scores increased from 26% pre-training to 74% at 16 weeks after training; 20 weeks after training, these improvements increased to 95%. However, they found no significant changes in staff attributional dimensions but staff rated their knowledge levels as higher both immediately following training and 8 weeks later. O’Dwyer *et al.* (2017) reported that the behaviour support quality increased (statistically significant improvement compared to control group). However, the quality of behaviour support plans of the trained group remained below optimal levels. No significant change was reported with regards the use of chemical restraint. Reid *et al.* (2003) reported that 85% of staff successfully completed the training by performing all classroom and on-the-job skills checks at mastery criterion. All classroom probes were successful although frequently several observation and feedback sessions were required. Finally, Wardale *et al.* (2014) achieved positive results for support plan quality, significant change in causal attributions and staff knowledge, but no significant difference with evidence-based practices.

### Service user outcomes

Service user outcomes with regards to the reduction in challenging behaviour were reported in five studies (Crates and Spicer 2012, Dench 2005, MacDonald *et al.* 2018, McGill *et al.* 2018, O’Dwyer *et al.* 2017). Only three studies reported on changes in residents’ quality of life (Dench 2005, MacDonald *et al.* 2018, McGill *et al.* 2018) and one study reported on changes in mental health (O’Dwyer *et al.* 2017). Crates and Spicer (2012) demonstrated a statistically significant reduction of targeted challenging behaviour at 3-month follow-up for 27/30 service users. Dench (2005) reported that 56% cases presented with significant improvements with regards to challenging behaviour (defined by reduction to less than 30% of baseline) within three months of training. They argued that the Quality-of-Life Questionnaire (Schalock and Keith 1993) was insufficiently sensitive to measure some of the anecdotal evidence. MacDonald *et al.* (2018) achieved a significant reduction in challenging behaviour, however reported no significant increase for quality of life at 6-months follow up. McGill *et al.* (2018) reported that challenging behaviour reduced significantly more in the experimental group than in the control group and that the difference between these groups was maintained at follow-up. They found no significant difference between the groups in terms of quality of life. O’Dwyer *et al.* (2017) did not achieve a significant change for severity of challenging behaviour, however, they reported statistically significant change in client mental health.

### Other outcomes

Other outcomes that were not the focus of the present review, for example outcomes for staff knowledge, attitudes, or beliefs, were reported in 8 studies. Specifically, 4 studies reported on staff knowledge (Haberlin *et al.* 2012, MacDonald *et al.* 2018, McKenzie *et al.* 2002, Wardale *et al.* 2014), 3 studies reported on staff attitudes or beliefs, including challenging behaviour attribution and evidence-based practice attitude (MacDonald *et al.* 2018, McKenzie *et al.* 2002, Wardale *et al.* 2014); three studies reported the outcomes for the organization, including cost effectiveness, referral data, and quality of support (Crates and Spicer 2012, Dench 2005, McGdaill *et al.* 2018); one study reported the use of physical restraints (O’Dwyer *et al.* 2017); and one study focussed on practice leadership (MacDonald *et al.* 2018).

### Organisational behaviour management systems

Organisational behaviour management (OBM) strategies were considered according to strategies that were used; either antecedent-based (e.g. training of supervisors; allocation of responsibility to trainees; prompts; additional resources) and/or consequence-based (e.g. monitoring, feedback) (Table 3). Five studies evaluated the use of two antecedent OBM strategies (inclusion of supervisors in staff training and allocating responsibility to generalize learning) (Crates and Spicer 2012, Haberlin *et al.* 2012, MacDonald *et al.* 2018, O’Dwyer *et al.* 2017, Reid *et al.* 2003). McGill *et al.* (2018) used three antecedent strategies (including supervisors in training, prompts, and change in resource availability), while two studies (Dench 2005, Wardale *et al.* 2014) reported only one antecedent strategy (training supervisors). From those studies, only one (Wardale *et al.* 2014) did not report the use of consequence based OBM strategy (monitoring, feedback). McKenzie *et al.* (2002) did not describe any organisational supporting systems in place to assist the staff training outcomes.

**Table 3.** Organisational systems in combination with training.

Authors	Antecedent-based				Consequence-based			
	Assess	Super roles	Response	Others	Mon/feed systems	Others	Effectiveness	Maintenance/generalization
Crates and Spicer 2012		Second generation trainees: allied health or nursing professionals, individuals with degree qualifications or adult education qualifications working in leadership roles within government and nongovernment service provision organisations	First generation trainees were asked to train the second-generation trainees		PSR used for the intervention plans (as part of the longitudinal practicum)		**Staff:** Quality of Behaviour Assessment and Intervention Plans: positive Fidelity of Plans: negative **Residents**: Challenging behaviour: positive **Others:** Referrals: positive Cost effectiveness: anecdotal positive	**Generalization**: positive
Dench 2005		Supervisors of trainees-staff are attending a supervisors’ workshop			PSR used by supervisors		**Staff:** Quality of Behaviour Assessment and Intervention Plans: positive Fidelity of Plans: no information **Residents**: Challenging behaviour, positive Quality of life: no information (only anecdotal) **Others:** Organisational cost benefit analysis: positive	
Haberlin *et al.* 2012		4 supervisors in one group	Pyramidal training (train the trainer program) for one of the groups		Training supervisors on how to deliver feedback		**Staff** Correct teaching procedures: positive for both groups (higher results for pyramidal group) Knowledge: positive for both groups	**Generalization:** Positive **Maintenance:** Positive for pyramidal group only
MacDonald * et al.* 2018		72 managers first level managers	Manage and review staff practise was one of the objectives of the course		Training in PSR		**Staff** Quality of staff support Fidelity of PBS plans and staff performance: negative **Knowledge**: positive for the experimental group **Residents:** Challenging behaviour: positive Quality of life: Negative	**Generalization:** Positive **Maintenance:** Positive for pyramidal group only
McGill *et al.* 2018	Assessing provider’s score on defined social care standards	Managers and direct care staff were trained together by the researchers.		3 h briefing session	Using traffic light system to evaluate performance (researchers faded their support and managers carried on the responsibility)		**Staff:** Quality of support: Positive (ASM scores) Negative (Mean percentage of engagement) **Residents:** Challenging behaviour: positive Quality of life: negative **Others:** Provider’s quality of support: positive	**Maintenance**: positive for reduction in challenging behaviour
McKenzie *et al.* 2002							**Staff:** Staff practice: positive Knowledge: positive **Others:** Staff challenging behaviour attribution: negative	**Maintenance**: positive for staff practice and staff knowledge
O’Dwyer *et al.* 2017		Staff at a team leader level	Staff responsible to create an implementation plan for the team, after the training.		Training for using BSP QE-II		**Staff**: Behaviour Support Plan quality: positive but the quality of the BSPs of the trained group remained below optimal levels **Residents**: Challenging behaviour: negative Mental health: positive **Others**: frequency of restraint: negative	**Generalization**: positive Change (quality of BSPs Remained below optimal Levels)
Reid *et al.* 2003		All trainees were supervisors	Through teaching supervisors feedback skills		Supervisors were taught feedback skills		**Staff:** Performance based training skills: positive	**Generalization**: the Impact on the trainees of The supervisors was not Measured
Wardale *et al.* 2014		25.9% of the sample were allied health professionals (psychologists, occupational therapists), 24.6% direct support workers, 22.4% service managers, 20.6% service coordinators or team leaders 6.6% ‘other’ (such as educational staff).					**Staff**: PSP quality: positive Knowledge: positive **Others:** Causal attributions: positive Evidence-based practises: negative	

Training staff for their supervisory roles generally is considered part of a system-wide staff training process (Reid *et al.* 2003) and this was recorded in all studies, except McKenzie *et al.* (2002). Wardale *et al.* (2014) did not use this as inclusion criteria for their study, but reported on multidisciplinary staff (i.e. allied health professionals, direct support workers, and team leaders) who had attended the training. Dench (2005) was the only study that did not specifically target staff in supervisory roles, while McGill *et al.* (2018) was the only study to describe training for both managers and direct care staff.

The responsibility to generalize training outcomes within the team was described in five studies in different ways. In two studies, the ‘train the trainer’ model was assessed (Crates and Spicer 2012, Haberlin *et al.* 2012), while two of the studies (MacDonald and McGill 2013, O’Dwyer *et al.* 2017) described practice-based training, where the individuals in supervisory roles had the responsibility to ensure generalisation of new skills across the team. Reid *et al.* (2003) was the only study that measured supervisor competency to train font-line staff in specific skills.

Consequence-based systems that included monitoring and feedback were reported in seven of the studies and this included monitoring performance, evaluating intervention fidelity, or mentoring regarding specific staff behaviours as part of maintenance procedures. For example, Macdonald and McGill (2013) included PSR in their manager training to ensure ongoing evaluation regarding the organisation of team meetings and monitoring staff performance via direct observations. The results of the PSR were presented to the team with graphs to deliver visual feedback. O’Dwyer *et al.* (2017) used the BSP QE-II, in order to assess the quality of behaviour support plans, including staff communication about their implementation. McGill *et al.* (2018) used a traffic light system to monitor progress. Although initially the feedback to the managers was completed by the researchers, in the end their support was faded, and managers acquired this new responsibility. There was no detailed description exactly how staff training was conducted.

Another approach to monitoring performance and delivering performance-based feedback was reported by Reid *et al.* (2003), who trained supervisors to conduct evaluative observations and provide feedback and performance-based staff training. Haberlin *et al.* (2012) instructed the pyramidal training group of supervisors to deliver performance-based feedback while the direct care staff group did not receive this training. Finally, Dench (2005) used a quality assurance process with responsibility for supervisors of the trainees.

Only two studies reported on OBM outcomes. Crates and Spicer (2012) found that the priority of referrals reduced from 100% Priority 1 in the first year of training to 25% after training. McGill *et al.* (2018) reported that the quality of service improved and achieved a median percentage after training of 80.1% in each setting.

The two studies (Crates and Spicer 2012, Haberlin *et al.* 2012) that applied a pyramidal ‘training the trainer’ model described at least one positive target outcome for staff. Crates and Spicer (2012) found that first-generation trainees were able to successfully train second-generation staff to produce good quality reports. The study also demonstrated positive outcomes for residents, including the reduction of challenging behaviour and anecdotal evidence of improvements of quality of life. A decrease in severity rating was reported also as a positive outcome for the organisation.

Haberlin *et al.* (2012) compared the outcomes of ‘train the trainer’ with group training of staff. The group-based training led to positive staff outcome, although the pyramidal training was more positive for all staff outcomes, including staff knowledge, practical skills, and maintenance of learning. In a similar cascade approach described by Macdonald *et al.* (2018), first level managers were trained and the impact on this training was assessed for the managers, their staff teams, and their service users. However, the training did not demonstrate significant change in any of their behaviour, although there was an increase in manager’s knowledge and a reduction of residents’ challenging behaviour. No significant change was reported for residents’ quality of life.

Other studies that used supervisor training reported less effect. For example, O’Dwyer *et al.* (2017) assessed the impact of training supervisors, who then were asked to communicate their learning to their team. Although, compared to the control group, statistically significant improvements were reported regarding the quality of behaviour support plans and their implementation, these findings remained below optimal levels. Furthermore, the authors did not report significant change in the use of chemical restraint or the severity of challenging behaviour, but they reported significant change in client’s mental health. Reid *et al.* (2003) focused on supervisor competence rather than the effect on other staff and reported that, after training, 85% of the supervisors successfully performed all on-the-job skills checks.

McGill *et al.* (2018) was the only study that used more than two antecedent-based strategies. They described a setting-wide intervention, using a cluster randomized controlled trial that included a briefing meeting with the staff and management team, an assessment of provider needs, including staff training needs, additional resource by having researcher conducting the intervention, and a traffic light system to monitor staff performance. The quality of care improved for the training group and challenging behaviour of residents reduced significantly. These improvements were maintained at follow up. However, compared with the control group, residents’ quality of life did not improve significantly.

The only organisational system used by Wardale *et al.* (2014) was to include trainees in supervisory roles along with other staff with the results that staff causal attributions and staff knowledge changed significantly but there was no significant difference regarding attitudes to evidence-based practices.

McKenzie *et al.* (2002) did not report any organisational systems to support staff training. Their training consisted of a one-day course regarding challenging behaviour. They reported positive outcomes for maintenance of staff knowledge and practice skills, as staff self-scored their knowledge as higher both immediately after training and eight weeks later. These perceptions were confirmed 16 weeks after training when the PCR score had increased from 26% to 74% and 20 weeks after training when the score had increased to 95%.

### Social validity

Social validity was reported by Crates and Spicer (2012), staff satisfaction was assessed by Dench (2005) and Haberlin *et al.* (2012), training was evaluated by McGill *et al.* (2018) and Wardale *et al.* (2014), and training acceptability was assessed by Reid *et al.* (2003). Findings were positive for all of these studies.

Crates and Spicer (2012) used the Social Validity Survey (SVS; LaVigna *et al.* 2005) and found that the total mean for all SVS item scores was 88.3% (range of 69.2%-98.5%); for their 2006 Tasmanian cohort the mean score was 89.8% (range 77%-97%); for the 2008 Tasmanian cohort the mean score was 84.8% (range 76%-94%). Dench (2005) assessed staff satisfaction of staff and found that 94% of staff rated the course as ‘very relevant’. Haberlin *et al.* (2012) used a satisfaction survey and reported positive results for both groups, with higher scored for the consultant-led training. McGill *et al.* (2018) used a one-page questionnaire for the overall evaluation that was completed by staff, family members professionals and found overall positive ratings. Reid *et al.* (2003) used an acceptability questionnaire and got positive results with 95% of trainees reporting the training to have been extremely or very useful. Finally, Wardale *et al.*’s (2014) evaluation questionnaire also produced positive results.

### Quality assessment of the studies

The overall quality of the studies was assessed using Reichow *et al.*’s (2008; cf., Reichow *et al.* 2011) evaluative method for determining evidence-based practices (EBP) in autism (Table 4). One study was rated as ‘strong’ (McGill *et al.* 2018) and one as ‘adequate’ (Macdonald *et al.* 2018). These studies reported good interobserver agreement (IOA), good levels of generalization and maintenance, and high levels of social validity. McGill *et al.* (2018) also reported randomization, attrition, and effect size.

**Table 4.** Quality assessment according to Reichow *et al.* (2011).

	Essential quality indicators						Desirable quality indicators								
	PART	IV	CC	DV	LRQ	STAT	RA	IOA	BR	FID	ATR	G/M	ES	SV	Quality of study
Crates and Spicer 2012	U	H	U	H	H	H	No	No	No	No	No	No	Yes	Yes	Weak
Dench 2005	U	A	U	A	H	U	No	No	No	No	No	No	No	No	Weak
Haberlin *et al.* 2012	U	H	H	H	H	A	No	Yes	No	No	No	Yes	Yes	Yes	Weak
Macdonald *et al.* 2018	H	H	A	H	H	H	No	Yes	No	No	No	Yes	No	Yes	Adequate
McGill *et al.* 2018	H	H	H	H	H	H	Yes	Yes	No	No	Yes	Yes	Yes	Yes	Strong
McKenzie *et al.* 2002	A	A	U	A	H	H	No	No	No	No	No	Yes	Yes	Yes	Weak
O’Dwyer *et al.* 2017	H	H	U	H	H	H	No	No	No	No	No	No	Yes	Yes	Weak
Reid *et al.* 2003	U	H	H	H	H	U	No	Yes	No	No	No	No	Yes	Yes	Weak
Wardale *et al.* 2014	A	H	U	H	H	H	No	Yes	No	No	No	No	Yes	Yes	Weak

The remaining seven studies were rated as ‘weak’ due to an ‘unacceptable score’ for at least one of the primary indicators. Five of these studies failed to report a comparison condition (Crates and Spicer 2012, Dench 2005, McKenzie *et al.* 2002, O’Dwyer *et al.* 2017, Wardale *et al.* 2014), while four studies did not provide adequate information for participants characteristics (Crates and Spicer 2012, Dench 2005, Haberlin *et al.* 2012, Reid *et al.* 2003), and two studies failed to include statistical analysis (Dench 2005, Reid *et al.* 2003). Furthermore, these studies received a negative score for at least four secondary indicators. None of those studies reported randomization for group allocation, raters being blinded to the treatment condition, procedural fidelity, or criteria for attrition. Two of these studies provided information for generalization and/or maintenance (Haberlin *et al.* 2012, McKenzie *et al.* 2002), three studies reported interobserver agreement (IOA; Haberlin *et al.* 2012, Reid *et al.* 2003, Wardale *et al.* 2014), and all of them reported social validity. Finally, acceptable effect size was reported in all those studies, expect in Dench (2005).

## Discussion

The present review identified nine studies that reported results of staff training in the general PBS framework and/or the whole science approach of ABA and analysed these studies according to a) general study and training information, b) type of outcomes and measurement systems, c) organisational behaviour management (OBM) systems in place related to staff training, d) effectiveness of the training, and e) quality of the studies. Although the variety of training methods, OBM systems, and outcome measures make a direct comparison between studies difficult, overall, training appeared to have a positive effect on staff knowledge and practice skills, especially with regards to developing and implementing behaviour support plans.

Interestingly, only few studies included specific behavioural resident outcomes or more general residents’ quality of life measures. They also lacked focus on stakeholder outcomes. This lack of focus on residents was particularly conspicuous in the context of PBS claims regarding person-centred processes, values, and disability rights as well as stakeholder involvement, where one would expect research to be particularly focussed on outcomes for residents and stakeholder. The studies that reported positive outcomes considered mainly a reduction in challenging behaviour. Measurable improvements with regards to quality of life were not demonstrated, although it was not clear whether this was due to inclusion criteria in this review, or the difficulties in measurement, lack of attention to detail, or lack of progress. Clearly, this gap in the research literature should be addressed in future studies reporting more precisely on behavioural outcomes for residents as well as stakeholders.

A number of OBM processes were utilised in the studies. These included antecedent as well as consequence-based procedures. Generally, the pyramidal ‘train the trainer’ systems seemed to work well, but a number of questions remained. For example, it was not clear, if those responsible for training second-generation staff were given specific instruction to do so, or equally, if the second-generation staff were expecting to learn from those who had received the initial training. None of the studies described replicable procedures for this cascading process. Future research should explore these issues as findings would be of interest of researchers in OBM more generally, who intend to use the pyramid system to train their staff (Dillenburger 2016). The question is whether or not untrained staff learn from trained staff without added instruction to do so (e.g. model learning; Cooper at al. 2019), or if specific procedures may be necessary to ensure pyramidal learning takes place (Daniels 2000, Daniels and Bailey 2015). While other antecedent and consequence-based OBM strategies were used, the description of those systems also was not always clear enough to allow replication. Given the growing pool of OBM research especially in relation to occupational safety (Agnew and Uhl 2021), it would be important in future studies that procedures are described in enough detail to allow for replication.

With regards to the evaluation of the strength of the studies, it is important to remember that this kind of research is conducted in residential settings were the strict criteria, such as randomisation and blinding commonly are impossible to achieve (Keenan and Dillenburger 2011). Other criteria, such as detailed description of diagnostic processes, also often are not available in applied studies of adults in residential care, given that most of these residents would have been diagnosed as children and entered residential care as adults (Dillenburger and McKerr 2011). As such, their exact diagnostic history often remains unknown. Therefore, the categorisation of the studies included in this review should be viewed cautiously. A more flexible categorisation system would likely conclude that most of these studies are strong enough to support the concept of staff training in residential care for adults with intellectual disabilities and challenging behaviour. In fact, findings reported here largely confirm those reported in previous reviews (Gormley *et al.* 2020, MacDonald and McGill 2013, Shapiro and Kazemi 2017) and meta-analyses (van Oorsouw *et al.* 2009) that had concentrated on specific behavioural intervention procedures. Evidently, staff training in the PBS framework and/or training in the overarching science of behaviour analysis can have an impact on staff behaviour as well as residents’ outcome. Future studies would need to try and achieve stronger methodological rigour to ensure that the existing evidence gains in credibility.
